# The effects of cigarette smoking extracts on cell cycle and tumor spread: novel evidence

**DOI:** 10.2144/fsoa-2019-0017

**Published:** 2019-05-03

**Authors:** Aldo Pezzuto, Fabrizio Citarella, Ivana Croghan, Giuseppe Tonini

**Affiliations:** 1 Cardiovascular & Thoracic Department, AOU Sant’Andrea, Sapienza – Università di Roma, Roma, Italy; 2 Oncology Department, Campus Bio-Medico Università di Roma, Roma, Italy; 3 Department of Medicine Clinical Research Office & Primary Care Internal Medicine, Mayo Clinic, Rochester, MN 55905, USA

**Keywords:** apoptosis, autophagy, cancerization, carcinogenesis, cell cycle, cigarette smoke extracts, inflammation, invasion, metastasis, promotion

## Abstract

Cigarette smoking is a major preventable risk factor for lung cancer, contributing to lung cancer progression and metastasis. Moreover, cigarette smoking correlates with increased metastasis frequency of pancreatic, breast and bladder cancer. The aim of this review was to examine the role of cigarette smoke extract in cell cycle and cancer progression. Clinical impact and the effects of cigarette smoke extract on carcinogenesis are discussed. 98 of the over 5000 chemicals in tobacco smoke are known carcinogens that can act on cancer genes such as K-RAS and p53. Through various mechanisms these compounds can activate molecules involved in the cell cycle, such as cyclins, and molecules involved in apoptosis and autophagy, such as Beclin-1 or LC3B. A search of the literature, including *in vitro* and *in vivo* studies, was carried out and the results summarized.

## Abstract (executive summary)

There is evidence of cancerogenic effects of cigarette smoke compounds. Cigarette smoke extract is a tobacco condensate obtained by filtration processes. Studies have shown that it can modify the cell cycle, inducing uncontrolled cell proliferation. This effect occurs through activation of genetic and epigenetic pathways and increasing the expression of proteins involved in inflammation. The pathways activated by cigarette smoke extract open up opportunities for researchers to develop new targeted therapies toward the specific molecules involved. Furthermore, the effects exerted by cigarette smoke extract on normal epithelial cells hold potential for use in the development of prevention medicine and early cancer diagnosis.

Lung cancer is the leading cause of cancer death worldwide with an estimated 1.2 million new cases annually. A tight link between smoking and lung cancer development has been demonstrated and causes more than 1 million deaths annually [1] Tobacco has a complex make up, with over 5000 substances that can damage cell structures (including DNA, lipids and proteins) in both direct and indirect ways [2].

These carcinogens include tobacco-specific N-nitrosamines, polycyclic aromatic hydrocarbons (PAHs), aromatic amines, aldehydes, phenols, volatile hydrocarbons and nitro compounds. The emission level has been identified for more than 500 smoke chemicals and a toxicological threshold was established for 98 hazardous components. These are able to cause carcinogenesis, bronchial and cardiovascular injuries. Each of the aforementioned substances was able to elicit an upregulation of oncogenes and transcriptional factors inducing cancer [3]. Some receptors, such as α7 and β-adrenergic, activated by nicotine and its compounds are also responsible for cancer induction through the activation of cascade signals. These signals, such as cyclic-AMP and protein kinase A (PKA), in turn are able to increase DNA synthesis and cell proliferation 4. The expression of α7-nAChR in non-small-cell lung cancer (NSCLC) tissues has been shown to be higher in smoking patients with squamous carcinomas than those with adenocarcinomas [2–4].

Cigarette smoke is subdivided into two phases: the hydrophobic tar cigarette smoke condensate (CSC) or particulate fraction, and the hydrophilic or gas phase (cigarette smoke extract, [CSE]). CSE has been used in research seeking to understand the effects of smoking on cells *in vitro*, including apoptosis, inflammation, metastasis and proliferation [5].

In such studies, CSE is usually obtained using cigarettes containing 10 mg of tar and 1 mg of nicotine. Cigarettes are completely heated and combusted and mainstream smoke bubbled in RPMI-medium and then filtered, thus mimicking the action of smoke *in vivo* in humans.

The purpose of this review is to summarize research into the effects of CSE on cell culture and to look for possible application of these findings. For this purpose, we searched the literature using the Pubmed database.

## CSE & the cell cycle

CSE is obtained from normal mainstream cigarettes filtered and drawn in tubes containing phosphate buffer saline (PBS). The main molecules involved in cell cycle and carcinogenesis that are affected by CSE are summarized in Table 1.

**Table 1.** Main factors involved in cell cycle and inflammatory regulation affected by cigarette smoke.

Factors	Action	Ref.
β-catenin	Cell adhesion, injury repair	[5]
Gsk 3β	Contribution to energy metabolism	[7]
MTA1	Induction of metastasis	[9,10]
RBM5	Arrest cell cycle in G1/S phase	[12,13,14]
PLTP	Induction of TGF-β and CDK	[16,17,18]
P16	CDK inhibitor, arrest in phase G1	[20,21]
P21	CDK inhibitor	[22]
Inc RNA	Promotion on G1–S transition	[24]
OSGIN1	Induction of autophagy and apoptosis	[26,27]
Beclin 1	Pro-apoptotic function	[27,28]
Hsp72	Prevention of lung damage	[32]
PARG	Cell viability	[34]
GOLPH3	Fostering of cancer cell survival	[36]
NRF2-KEAP1	Protection from oxidative stress	[42,43]
hBD2	Immune-modulation	[44]
PLD	Regulation of inflammatory cytokine	[54,55]
CCAT1	Tumorigenic function	[58]

Gas chromatography–mass spectrometry (GC–MS) is usually used to find the concentration of a single component. Various *in vitro* studies have been conducted to explore the effects of CSE on genetic and epigenetic molecules. Tian *et al*. investigated the effects of CSE on the β-catenin/TCF pathway. β-catenin is a notorious mediator and regulator of the cell cycle, cellular adhesion and injury repair [5]; its accumulation in the nucleus may play a pivotal role in activating β-catenin/TCF signaling [6,7]. Conversely, Glycogen synthase kinase 3β (GSK3β) is a negative regulator kinase of β-catenin [8]. Nicotine and Nicotine-derived nitrosamine ketone (NNK), two main components of smoke, contribute to *in vitro* GSK3β phosphorylation [9–11]. The authors of this study co-cultured alveolar epithelial cell line (A549) in presence of CSE at different concentrations for 24 h. Cell viability lowered, at 2% concentration, proportionally to CSE concentration. After exposure to CSE, western blot using anti-GSK3β and antiphopshorylated GSK3β (Ser-9-GSK3β) showed a dose-dependent GSK3β decrease and a phopshorylated GSK3β increase. In the same way, a dose-dependent increase of β-catenin was observed both in the nucleus and in the cytoplasm. GSK3β overexpression was demonstrated to be able to prevent β-catenin transcription in CSE-exposed cells. Thus, impairment in β-catenin and GSK3β balance results is a potential mechanism involved in pulmonary damage resulting from a smoking habit [11].

CSE could influence the expression of another protein called MTA1 (metastases associated protein) [12], a mediator of tumorigenesis. This is a member of the nucleosome remodeling and deacetylating (NuRD) complex and acts influencing the acetylation status of chromatin. Xu and colleagues found a statistically significant difference (p < 0.05) in MTA1-expression between NSCLC cells (63.5%) and normal pulmonary cells (15.6%) [11]. A correlation between smoking and MTA1 expression was shown, consistent with other studies according to which MTA1 correlates with N positivity and TNM staging. MTA1 mRNA and protein expression were investigated *in vitro* by western blotting and RT-PCR; lung adenocarcinoma A549 culture cells displayed the lowest levels in comparison with lower metastatic LH cells, while higher metastatic BE1 cells had the highest levels. Exposure to CSE increased both cellular invasive ability and MTA1 expression. A significant correlation was found between the number of invasive cells and MTA1 protein expression (p = 0.004) and MTA1-mRNA (p = 0.008). MTA1 activity can be consequently considered as a smoking-induced marker of invasiveness [12].

Another factor tightly linked with tumor progression is the RNA-binding motif protein 5 (RBM5), whose gene is named LUCA-15 or H37, which is a direct modulator of cell cycle. Its downregulation has been shown to occur in primary lung cancer [13,14] and its expression is negatively correlated with smoking status [15]. It was demonstrated that *in vitro* exposure to CSE reduced RBM5 mRNA and protein levels both in human bronchial epithelial cells (BEAS-2B) and in cancerous cells (A549) [15]. Overexpression of RBM attenuates both proliferation and invasion of CSE-transformed BEAS-2B cells and reduces proliferation mediators such as hypoxia induced factor (HIF-1α), VEGF and matrix metalloproteinase (MMP-2). Interestingly, RMB5 is able to arrest the cell cycle at the G1/S phase in the CSE-transformed BEAS-2B cells. In fact, the authors pointed out an increase in p53 and p21, along with a reduction in CDK4, CDK6, cyclin D1 and cyclin A [16,17]. Higher levels of cleaved caspase-3, caspase-9 and BAX and lower levels of Bcl-2 confirmed the suspicion that high RMB5 is responsible for apoptosis of transformed BEAS-2B cells. Over-expression of RBM5 also managed to decrease tumor growth *in vivo*. In conclusion, RBM5 is a negative regulator of lung carcinogenesis and progression, but its specific role had heretofore never been investigated. We know that it is able to prolong G1 phase or influence the expression of several genes involved in cell proliferation, but, in addition, it is reasonable to think that it works as a positive regulator of p53, that in turn increases p21, inducing G1/S phase arrest [16].

Another protein whose expression is affected by CSE is phospholipid transfer protein (PLTP). This is an enzyme expressed by alveolar epithelial type II cells intervening in phospholipid trafficking. Plasmatic PLTP activity was higher in smokers than nonsmokers [18] and the protein is involved in CSE-related apoptosis [19]. *In vitro* rat lung cells exposed to CSE saw an increase in PLTP and TGF-β1 mRNA and protein levels, a decrease in Cyclin D1 and CDK4 and an arrest in G1 phase in the experiments led by Chai and colleagues [17]. Inhibition of TGF-β1 enhanced CDK4 and cyclin D1, but did not have any effect on PLTP, leading to the conclusion that TGF-β1 is a downstream mediator of PLTP and the effects of CSE on cell cycle depend on the PLTP/TGF-β1/Cyclin D1/CDK4 pathway.

One experimental study was carried out through the incubation of placental cell lines cultured in Dulbecco’s medium with CSE [20]. Western blot assay was applied to quantify the proteins involved in cell migration and invasion and to analyze the cell cycle process. A decreased level of proliferating cell nuclear antigen in the presence of CSE was shown.

A study showed an altered level of cathepsin D that was detected when investigating the carcinogenic effects of CSE in placental cells. The aforementioned protein is an endoprotease normally expressed on cancer cells. It was shown that its level is increased by CSE in a concentration-dependent manner [20]. CSE was able to affect several other proteins, such as human chorionic gonadotropin (hCG-β) protein in placenta-derived JEG-3 cells. Overall, these results indicate that exposure of placental cells to CSE deregulates the cell cycle. CSE inhibited growth factors and proliferation in human choriocarcinoma, while nicotine affects placenta cells proliferation.

Furthermore, CSE treatment promotes the viability and proliferation of human aortic smooth muscle cell (HAOSMCs) and decreases P16 protein expression in cancer cells [21]. P16 is a CDK inhibitor, which inhibits cyclin-dependent kinase CDK4 and CDK6 activation and their downstream retinoblastoma protein (Rb-E2F) signaling, thereby preventing cell cycle progression from G1 to S phase. Downregulation of P16 enhances CSE-mediated cell proliferation and cell cycle arrest in culture of HAOSMCs. The P16 promoter was hypermethylated in HAOSMCs in the presence of CSE, hindering its activity [21]. Thus, downregulation of p16 causes an alteration of G/S phase transition. This was reversed by overexpression of p16. As a consequence of p16 downregulation, a significant increase in the expression of CDK 4, CDK6 and phosphorylated retinoblastoma (p-Rb) protein is induced, resulting in a significant increase in the ratio of cells in S phase. The function of p16 was studied, including its significance and clinical value [22]. In summary, CSE could affect cell growth through the β1-cyclin D1-CDK4 pathway. CDK1 and cyclin D1 were increased after exposure.

Another gene which is involved in cell cycle modulation is the oncosuppressor p21, a cyclin dependent kinase inhibitor. The HOTAIR gene seems to be related to the presence of CSE [23]. Indeed, p21 (known as p21[WAF1/Cip1]) is one of the factors that promote cell cycle arrest in the G0 phase in response to a variety of stimuli. The inhibitory effect of p21 on cell cycle progression seems to correlate with its nuclear localization. P21 can be induced by stimulation in both p53-dependent and p53-independent pathways. Among its activities, p21 is able to modulate apoptosis. P21 can also play a role in DNA repair by interacting with proliferating cell nuclear antigen [24]

## Noncoding RNA

An altered cell cycle was also found associated with the silencing of miR-218, which is a noncoding RNA [25]. CSE caused an increase of CCAT1 levels and a decrease of miR-218 levels in human bronchial epithelial (HBE) cells. Another noncoding RNA (IncRNA) was examined in HBE cells treated with CSE at a dose of 2  μg/ml to establish a malignantly transformed cellular model. Long intergenic noncoding RNA (linc00152) serum level in CSE-exposed individuals was increased in a dose-dependent manner. It is involved in regulation of cell adhesion, epithelial transition and other malignant phenotypes, which in turn, affected metastasis *in vivo*. Moreover, linc00152 is able to promote cyclin D1 expression and G1/S transition [26].

## CSE-mediated autophagy & apoptosis

One smoke-associated factor is OSGIN1 which upon upregulation is able to induce apoptosis or autophagy. It is a gene involved in oxidative stress and cell death in other tissues; it was indeed markedly upregulated in smokers compared with nonsmokers in airway epithelium [27]. As a consequence, markers of inflammation and autophagy were found to be highly expressed. An increased level of markers of apoptosis such as caspase were also found in *in vitro* immunoblot assay analysis [28]

FAK activation was also induced by CSE through phosphorylation along with the activation of the unfolded protein response, which is a marker of cell death [28]. Upregulation of beclin-1 and downregulation of p63 indicates an autophagy cell process.

Another factor involved in autophagy and apoptosis that was found deregulated is microtubule-associated protein light chain (LC3B). It is a regulator of the apoptosis-extrinsic pathway engaged with the Fas complex. CSE exposure induces a rapid dissociation of LC3B from Fas and the activation of apoptosis signaling [29].

In a toxicological model of CSE exposure to epithelial cells, the cells die through the apoptosis-extrinsic pathway that involves the activation of Fas-dependent death-inducing signaling complex (DISC) and downstream activation of caspases 8, 9 and 3. CSE exposure increases autophagosome formation in epithelial cells. Knockdown of autophagy proteins Beclin-1 or LC3B could inhibit CSE-induced apoptosis [30]. Caspases are the primary drivers of apoptotic cell death, cleaving cellular proteins that are critical for dismantling the dying cell. Caspases are a family of endoproteases that provide critical links in cell regulatory networks.

The *in vitro* effects of CSE and steroids on Heat shock proteins, in particular Hsp72, was investigated by Gal *et al*. Heat shock proteins contribute to limitation of cellular injury and apoptosis [31]; in particular, the inducible form of Hsp70 (Hsp72) prevents many kinds of cellular damage [32]. Steroids are widely administrated in patients affected by respiratory diseases and their effects are Hsp regulated [33]. In the mentioned experiments, A549 cell apoptosis was increased by CSE, but prevented by co-treatment with dexamethasone (DEX). Conversely, DEX did not influence CSE-induced cellular necrosis. Furthermore, if exposure to both CSE and to DEX did not significantly lower Hsp72 mRNA, DEX could induce a significant increase of Hsp72 mRNA in CSE-exposed cells. CSE increased Hsp 72 expressing cells above all in the presence of DEX. Transfection with siRNA led to a reduction of Hsp72 expression in steroid-naive controls, in CSE-pretreated cells and in DEX+CSE-pretreated cells and a proportional consequent rise of apoptosis. The experiments show that Hsp70 activation in presence of steroid administration can play an anti-apoptotic role in smokers [34].

Going on to the modulation of factors involved in autophagy and apoptosis, it is noteworthy to highlight the effects of smoking on PARylation and dePARylation enzymes was studied for the first time by Kovacs. Poly ADP ribosylation – PARylation – has a regulatory role on apoptosis and necrosis in the presence of cellular damage. *In vitro* CSE induced an increase in PAR content in A549 cells, strengthened by PARG silencing but abolished by PARP-1 silencing. Interestingly, morphologic changes, loss of viability and plasmatic permeabilization due to CSE exposure were more evident in cell lines treated with lentiviral PARP-1 and PARG inhibitor vectors, with greater effects on the latter. CSE imbalanced the proliferative capacity too, with greater effect on PARG-inhibited cells. The authors demonstrated that PAR synthesis may be due to cellular superoxide and hydrogen peroxide production, rather than CSE directly. Furthermore, the lethal CSE effect did not seem to be due to typical apoptotic or necrotic pathways. Exposure to CSE in presence of inhibition of either PARP-1 or PARG indeed leads to higher cellular viability, survival and DNA-repairing unpaired processes. Thus, the PARP-1/PARG status can significantly influence CSE-related carcinogenesis [35].

Golgi phosphoprotein 3 (GOLPH3) is a protein of the trans-Golgi membrane and plays a certain role in cancer cell survival, differentiation and proliferation [36]. Yu and colleagues tested HBE cells *in vitro* with CSC. Exposure to CSC predictably lead to endoplasmic reticulum (ER) stress, as shown by high stress-related protein levels of Gro78/Bip, ATF6 and Ire-1α, and microscopic evidence of ER and Golgi damage [36,37]. The effect on Golgi apparatus was similar to the one observed after treatment with Tunicamycin that acts as an ER stressor and both fluorescence and western blot pointed out an increase of GOLPH3 in ER-damaged cells. In addition, CSE was responsible for impaired autophagy; western blot highlighted high concentrations of the autophagy-related proteins LC3, p62 and BECN1. In order to confirm GOLPH3 involvement after smoke exposure, western blot was performed *in vivo* on samples from NSCLC-diagnosed patients. This demonstrated high levels of GOLPH3 and mouse lungs exposed to NNK showed high titre in GOLPH3 mRNA and proteins. Even though smoking history was not significantly related to GOLPH3 increase in smokers [37], CSE can act through ER dysfunction and autophagy, and GOLPH3 behaves as a reliable marker of these cellular events [38,39].

## Tumorigenic effects of CSE

The effect of CSE was tested in lung cancer endothelial cells (ECs). Tumor-associated ECs play important roles in tumor angiogenesis and metastasis. Lung EC lines were used as a model to study the pathological effect of CSEs on human lung ECs, and it was found that a lower dose of 4% CSE caused abnormal morphological changes in ECs, increasing the permeability of the endothelial monolayer, whereas a higher concentration of 8% CSEs caused EC apoptosis [40]. CSE leads to an increase in the number of apoptotic cells and to a lesser extent to an increase in necrotic cells. CSEs induced approximately 11-fold overexpression of a protumorigenic IL-13 receptor α2 gene (IL-13Rα2) through the activation of protein kinase A (PKA) and cAMP response element-binding protein (CREB) signaling pathway [41]. The overexpression of IL-13Rα2 in lung ECs significantly increased the tumorigenic, migratory and angiogenic properties of cells, suggesting that IL-13Rα2 promotes the malignant transformation of lung ECs and genesis of tumor-associated ECs [39]. Its expression is closely related to cancer resistance and poor prognosis, too.

Another factor associated with poor prognosis is a SCAL 1 – its involvement in lung carcinogenesis was discovered by Thai and colleagues [42]. First of all, the authors examined RNA-expression in noninvasive lung cancer cell lines CL1-0 and in invasive lung cancer cell lines CL1-5: the lncRNA *SCAL1* was found higher in the latter group. A significant difference in XLOC expression between smokers and nonsmokers was found in two public RNA-seq datasets, suggesting that *in vivo* smoking is able to increase SCAL1 [42]. CSE *in vitro* exposure was then performed in HBE and NHBE cells and a higher expression of SCAL1 was highlighted. SCAL1 was demonstrated to be regulated by NRF2 (nuclear factor erythroid derived) and KEAP1 (Kelch-like ECH-associated protein 1). The NFR2-KEAP1 pathway is a well-known pathway involved in cellular defense from oxidative stress [43,44]. Going into depth, siRNA knockdown of NRF2 in A549 cells and in CSE-exposed HBE1 cells containing high levels of SCAL1 significantly reduced the levels of the mentioned lncRNA. At the same time, KEAP1 inhibition increased SCAL1 in CSE non exposed HBE1 cells. Moreover, SCAL1 knockdown effects were reported in terms of a reduction of cellular viability occurring in a direct-proportional relation to CSE concentration. As a consequence SCAL1 was defined as a key mediator of NFR2 activity in cellular protection [42].

Other key-regulators of the cell cycle are β-defensins (hBD2), which are mediators involved in immune-modulation during lung injury [45]. There is some evidence that hBD2, whose expression is enhanced by smoking, is able itself to contribute to toxic smoking effects [46,47]. Pierson *et al*. investigated the levels of β-defensins after CSE treatment. At the same time hBD3, hBD5 and hBD9 mRNA levels significantly increased after *in vitro* A549 cell exposure to both CSE and IL-1β, suggesting a common regulating mechanism. Smoke extract also induces the release of IL1-β that was used in control cells, since it is a stimulating mediator of β-defenins [48]

The function of histone methyltransferase EZH2, which has been demonstrated to be involved in hepatocellular cancerization [49], was studied *in vitro* as a potential epigenetic promoter in HBE treated with CSE. It catalyzes histone H3 lysine 27 trimethylation (H3K27me3) and influences miRNA regulation. CSE exposition reduced miR-218 expression in HBE cells, while increasing the levels of the oncogene BMI1 [50]. MiRna-218 downregulation correlates with cellular proliferation and apoptosis [51]. CSE increased expression of EZH2 and methylation of histone H3; epigenetic silencing of pre-miR-218 downregulation by EZH2 was discovered to be the molecular mechanism allowing increased expression of BMI1, whereas overexpression of miR-218 resulted in loss of cancer stemness properties and decrease of migration and invasion in cells treated with CSE. Thus miR-218 can be considered a negative regulator of cell malignant transformation [52].

## Smoke-induced inflammation & cancer

Regarding inflammation, CSE are known to induce expression of many interleukins, which are responsible for lung injury and carcinogenesis. Koo and Han investigated the role of PLD (phospholipase-D) in CSE-mediated inflammation [53]. *In vitro* exposure to CSE was found to increase IL-6, PLD and PLD activity in human bronchial epithelial cells (BEAS-2B); IL-6, as well as IL-8 [54] and Il-13 [55], are regulated by PLD too. In particular, PLD-1 isozyme silencing abrogated IL-6 expression. PLD activity is regulated mainly by Gi protein/PLC/PKC playing a role in inflammation [53,56]. Inhibition of Gi, PLC, PKC and ERK decreases PLD1 and IL-6 expression. CSE can indeed induce IL-6 expression by Gi/PLC/PLC7p38/PLD1 sequential activation, even though alternative PLD1 activating pathways are supposed to participate [57].

Other oncogenic factors whose expression is affected by smoking habit are c-Myc and CCAT1. C-Myc is one of the oncogenic transcription factors related to lung carcinogenesis [58], while CCAT1 is an lncRNA that has been demonstrated to be downregulated in lung cancer [59] Lu *et al*. initially noticed that *in vitro* increase of c-Myc and CCAT1 is proportional to CSE exposition.

C-Myc works as an inductor of CCAT1 in CSE-exposed HBE cells by binding to its promoter. Its activation could be inhibited by let-7c mi-RNA increase. CCAT1 creates a positive feedback by binding free let-7c, thus inducing higher levels of c-Myc, paving the way for a potential carcinogenic mechanism [60].

Other molecules are influenced by smoking, such as TGF-β1 produced in inflammatory conditions, which is involved in lung cancer metastasis and s increased *in vitro* by CSE exposition [61]. Smad2, a TGF-β1 mediator and MMP3, which is a key enzyme involved in metastasis, were induced *in vitro* in A549 cells treated with CSE [62]. In the study presented by Liao *et al*. TGF-β1, MMP3, Smad2 and invasive cells were reduced after exposure to a blocker of TGF-β1, showing that TGF-β1 and MMP3 are main actors in lung cancer invasiveness and share a potentially procarcinogenetic pathway [63].

## Conclusion

Cigarette smoking is the greatest and most preventable risk factor for cancer. Carcinogens present both in particulate and gas phases in smoke are able to promote tumor growth and metastasis by acting on the cell cycle, inducing epithelial–mesenchymal transition, invasion, induction of angiogenesis and influencing apoptosis and autophagy. CSE could induce cell transformation through different mechanisms acting on genetic and epigenetic factors. Direct and indirect smoke-induced mechanisms are recognized to act on cell growth and tumor invasiveness by deregulation of oncogenes or anti-oncogenes, altering the cell death process, and favoring inflammation and malignancy.

We reported a series of influential studies, mostly published in the last 5 years that, despite being heterogeneous, are useful in the comprehension of the several mechanisms and pathways involved in smoke-induced lung carcinogenesis, invasion and metastasis.

## Future perspective

CSE reproduces *in vitro* the action of chemicals compounds from smoke in humans *in vivo* and provides an opportunity to find new factors responsible for cancer growth and proliferation. This will allow scientists to study new pathways and discover factors potentially targetable with new therapies. Genetic and epigenetic processes should be studied further, and inflammatory markers could be counteracted by anti-oxidant natural or synthetic molecules suitable for cancer prevention.

**Executive summary**

- Cigarette smoke contains more than 5000 recognized substances.
- Smoking-induced carcinogenesis is a result of genetic mutation or the activation of protein receptors that in turn activate kinases responsible of cell growth and uncontrolled proliferation.
- Cigarette smoke extracts (CSEs) are obtained by filtration of cigarette smoke condensate.
- Many effects have been shown *in vitro* for both cancer and epithelial cells. Nicotine, a CSE compound, induces phosphorylation of GSK kinase favoring the action of proteins (β-catenin) that dysregulate the cell cycle. The expression of several is higher *in vitro*, such as MTA, which is responsible for cell growth through increased DNA synthesis.
- Other protein modulators of the cell cycle are altered by CSE leading to downregulation of cell cycle and inhibition of apoptosis. For example, through the inactivation of anti-oncogenes such as p16 and p21 another pathway occurs through which CSE induces carcinogenesis and cell proliferation inducing imbalance in the G1/S phase of the cell cycle.
- Noncoding RNAs are also influenced by CSE, in particular linc00152, promoting G1/S transition and metastasis.
- Autophagy and apoptosis of normal epithelial cells are CSE induced; in fact, proteins involved in those mechanisms are upregulated. Inflammation is also involved and cell adhesion altered.
- Inflammatory proteins such as HSP are increased by CSE and promote epithelial cell death. Conversely, proteins involved in DNA repair are downregulated and inhibited. Increased autophagy has been shown to be associated with the inhibition of factors that foster cancer cell survival.
- CSE is able to affect endothelial cell function and survival. The increased level of IL-13 suggests a potential stimulation of the so-called neo-angiogenesis. Genetic pathways evoked by CSE hinder the action of factors counteracting oxidative stress. Increased IL-1 alters the immune response by acting on defensin.
- Mediators of inflammation may be responsible for increased immune cell recruitment, and increase in reactive oxygen species. TGF-β, IL-6 and IL-8 are smoke-induced inflammatory mediators prone to promoting carcinogenesis.
